# In silico comparative analysis of glycoside hydrolase (GH) family 10 endo-(1-4)-beta-xylanase genes from *Eucalyptus grandis* and *Arabidopsis thaliana*

**DOI:** 10.1186/1753-6561-5-S7-P168

**Published:** 2011-09-13

**Authors:** Ritesh Mewalal, Desre Pinard, Eshchar Mizrachi, Alexander A Myburg

**Affiliations:** 1 Department of Genetics, Forestry and Agricultural Biotechnology Institute (FABI), University of Pretoria, Pretoria, 0002, South Africa

## Background

The hemicellulose xylan constitutes the major non-cellulosic component of plant secondary cell walls. It has been shown that xylan adsorbs to cellulose fibres and also covalently binds a carbon moiety of lignin [1,2]. *Eucalyptus* is an important hardwood tree genus used in the pulp and paper industry and has potential as biofuel feedstock. Xylan removal is expensive and uses environmentally harsh chemical treatments [3]. Previous studies have shown that endo-(1-4)-β-xylanase enzymes belonging to glycoside hydrolase (GH) family 10 internally attacks the xylan backbone resulting in shorter xylo-saccharide chains [4]. The recently sequenced *Eucalyptusgrandis* genome (DOE-JGI, http://www.phytozome.net) provides a unique opportunity to analyze the native endo-(1-4)-beta-xylanase proteins involved in xylan modification in eucalypt fibre cell walls. Detailed knowledge of endogenous xylanolytic enzymes from *Eucalyptus* could facilitate the development of strategies to enhance the processing of woody biomass for cellulose and biofuel production. The aims of this study are to identify xylem secondary cell wall-related endo-(1-4)-β-xylanase genes in the *E. grandis* genome and to perform a comparative analysis of the *Eucalyptus* xylanasepeptide sequences with those of previously studied *Arabidopsis* orthologs to provide a framework for assigning function to the *Eucalyptus* enzymes.

## Results

**Figure 1:**
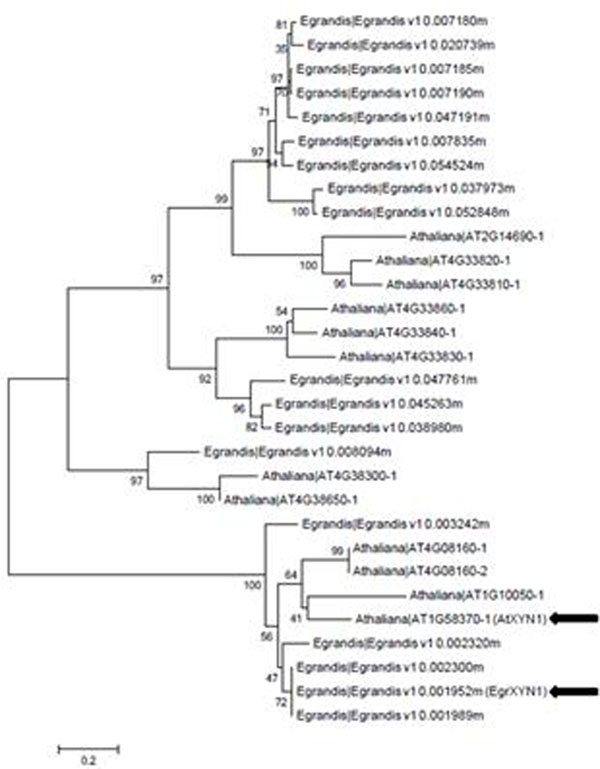
Co-phylogenetic analysis of the predicted protein sequences of GH10 family members (E score < 1e^-10^) in *E. grandis* and *A. thaliana.* Neighbor-joining and 1000 bootstrap replicates conducted in MEGA5. EgrXYN1 refers to Egrandis_v1_0.001952m while AtXYN1 refers to At1g58370 (indicated with bold arrows).

Analysis of the *E. grandis* genome sequence on Phytozome v7.0 (http://www.phytozome.net) for putative endo-(1-4)-β-xylanase genes resulted in the identification of 18 putative GH10 family members. The expression profile of each family member was assessed (via mRNA-Seq analysis, http://eucspresso.bi.up.ac.za/) to identify members with putative roles in xylem secondary cell wall metabolism. Egrandis_v1_0.001952m (designated *EgrXYN1*) showed the highest xylem to phloem and xylem to leaf expression ratios of the expressed *E. grandis* GH10 genes [5]. BLAST analysis (<1e-10) of the *A. thaliana* genome for putative orthologs to *EgrXYN1* and co-phylogenetic analysis of all 18 *E. grandis* enzymes with the putative *A. thaliana* xylanases revealed that *AtXYN1* (At1g58370) [4] was one of the closest putative orthologs to *EgrXYN1* (Figure 1). Alignment of the predicted amino acid sequences of *EgrXYN1* and *AtXYN1* Jalview 2.6.1 revealed 68.76% identity between the two sequences.

**Figure 2:**
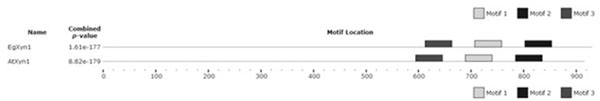
Mining of EgrXYN1 and AtXYN1 for novel motifs using MEME resulted in three additional motifs [6].

*In silico* biochemical analysis predicted that EgrXYN1 has a molecular weight of 103 kDa with a pI of 6.08. This is very similar to AtXYN1 which is 102 kDa with a pI of 6.1. The protein domain view in Phytozome (http://www.phytozome.net) revealed that EgrXYN1 contains three successive N-terminal β-sandwich carbohydrate binding modules IV (at amino acid positions 53-185, 216-357 and 387-532) which were also observed in AtXYN1. A protein motif search (http://motif.genome.jp) revealed that EgrXYN1 also contained a conserved and identical C-terminal GH10 active site sequence “GLPIWFTELDV” at amino acid position 802-812. Finally, *de novo* motif search of both AtXYN1 and EgrXYN1 using MEME revealed the presence of three additional novel C-terminal motifs present within both enzymes (Figure 2).

## Conclusion

The *E. grandis* genome contains 18 putative GH10 family members (at a BLAST threshold of 1e^-10^). One of these, *EgrXYN1* is highly preferentially expressed in *Eucalyptus* xylem tissues and shows highest similarity to *AtXYN1.* The similarities between AtXYN1and EgrXYN1 suggest similar biochemical properties and biological functions. Previous studies showed that AtXYN1::eGFP localized to the cell wall providing support for its function in cell wall modification. AtXYN1prom::GUS constructs expressed predominately in the vascular bundles suggesting that AtXYN1 (and therefore putatively EgrXYN1) is involved in secondary cell wall modification [4,7]. Future work will involve experimental validation of the biochemical properties and enzyme kinetics of EgrXYN1.
